# Calcium Dyshomeostasis Alters CCL5 Signaling in Differentiated PC12 Cells

**DOI:** 10.1155/2019/9616248

**Published:** 2019-03-26

**Authors:** Tomasz Radzik, Tomasz Boczek, Bozena Ferenc, Maciej Studzian, Lukasz Pulaski, Ludmila Zylinska

**Affiliations:** ^1^Department of Molecular Neurochemistry, Medical University, 6/8 Mazowiecka Str., 92-215 Lodz, Poland; ^2^Department of Ophthalmology, Stanford University School of Medicine, 1651 Page Mill Road, Palo Alto, 94304, USA; ^3^Department of Molecular Biophysics, University of Lodz, 12/16 Banacha Str., 90-237 Lodz, Poland; ^4^Laboratory of Transcriptional Regulation, Institute of Medical Biology PAS, 106 Lodowa Str., 93-232 Lodz, Poland

## Abstract

**Background:**

Plasma membrane Ca^2+^-ATPase (PMCA) is the most sensitive cellular calcium detector. It exists in four main isoforms (PMCA1-4), among which PMCA2 and PMCA3 are considered as fast-acting neuron-specific forms. In the brain, PMCA function declines progressively during aging; thereby impaired calcium homeostasis may contribute to some neurodegenerative diseases. These destructive processes can be propagated by proinflammatory chemokines, including chemokine CCL5, which causes phospholipase C-mediated liberation of Ca^2+^ from endoplasmic reticulum by IP_3_-gated channels.

**Methods:**

To mimic the changes in aged neurons we used stable transfected differentiated PC12 cells with downregulated PMCA2 or PMCA3 and analyzed the effect of CCL5 on calcium transients with Fluo-4 reagent. Chemokine receptors were evaluated using Western blot, and IP_3_ receptors expression level was assessed using qRT-PCR and Western blot.

**Results:**

In PMCA-reduced cell lines, CCL5 released more Ca^2+^ by IP_3_-sensitive receptors, and the time required for Ca^2+^ clearance was significantly longer. Also, in these lines we detected altered expression level of CCR5 and IP_3_ receptors.

**Conclusion:**

Although modification of PMCAs composition could provide some protection against calcium overload, reduction of PMCA2 appeared to be more detrimental to the cells than deficiency of PMCA3. Under pathological conditions, including inflammatory CCL5 action and long-lasting Ca^2+^ dyshomeostasis, insufficient cell protection may result in progressive degeneration and death of neurons.

## 1. Introduction

Growing body of evidence suggests that disrupted calcium homeostasis plays a detrimental role in triggering neurodegeneration. This process can also be propagated by repeated inflammatory reactions, including local production of chemokines. These events intensify particularly during aging, when the proper response to extracellular signals is decreased due to accumulation of multiple cellular damage and pathologies [1–4]. Injured cells are exposed to a prolonged elevation of intracellular Ca^2+^ that in turn initiates a number of abnormal processes, which can finally lead to cell death [5–7]. Disturbances in calcium homeostasis have been attributed to imbalance between calcium “on” and “off” systems, which affects cell survival. In healthy cells, the first step in decreasing cytosolic Ca^2+^ relies on three modes: uptake into endoplasmic reticulum by sarco/endoplasmic Ca^2+^-ATPase (SERCA), extrusion by high-capacity but low-affinity Na^+^/Ca^2+^ exchanger (NCX), and removal by plasma membrane Ca^2+^-ATPase (PMCA) [8, 9]. The latter is the most sensitive element with low capacity, but very high affinity. The enzyme is represented by 4 main isoforms with ~30 variants that exhibit differential spatial and developmental expression pattern [10, 11]. The two ubiquitous isoforms, PMCA1 and PMCA4, are far less effective in controlling calcium homeostasis than the two neuron-specific PMCA2 and PMCA3 isoforms. The expression profile of PMCAs changes significantly during development, reflecting the specific function of each isoform. Changes in PMCA expression and activity have also been reported during aging. It is believed that PMCA loss may significantly impair calcium extrusion in senescence neurons making them more susceptible to neurotoxic insults [12–17]. Ca^2+^-mediated neurotoxicity has been shown for several neurodegenerative diseases including Alzheimer's disease (AD), Huntington disease (HD), spinocerebellar ataxias (SCAs), Parkinson's disease (PD), schizophrenia, or bipolar disorder [6, 15, 18–22].

Additional factors contributing to neuronal death are inflammatory mediators, including some chemokines [1, 23]. Among 50 discovered chemokines, chemokine C-C motif ligand 5 (CCL5, RANTES) is of particular interest due to its potential role as a modulator of cellular metabolism and brain architecture [23–25]. CCL5 is constitutively expressed in the adult central nervous system, with region-specific expression pattern [26]. A remarkable increase of CCL5 in central nervous system (CNS) can be detected during permeabilization of the blood–brain barrier and after extensive production of CCL5 from astrocytes and microglial cells, triggered by proinflammatory factors [27–31]. One of the mechanisms of action of CCL5 is a positive control of cytosolic Ca^2+^ mobilization after binding to three receptors: CCR1, CCR3, and CCR5 [23, 32]. They are cell surface–associated, immune-regulatory G protein–coupled receptors (GPCRs). CCL5 binding activates a G protein, which subsequently activates phospholipase C (PLC) involved in a second messenger system. PLC-mediated hydrolysis of phosphatidylinositol 4,5-bisphosphate (PIP_2_) gives rise to two products: 1,2-diacylglycerol and inositol 1,4,5-triphosphate (IP_3_). IP_3_ stimulates the release of Ca^2+^ from intracellular stores through IP_3_ receptors, which exist in three different isoforms [33–35].

The present study was undertaken to clarify the potential role of CCL5-mediated signaling using the model of differentiated PC12 cells, which is one of the most frequently used models for studying neuronal processes. We have previously developed stable transfected lines of PC12 cells with downregulated expression of neuron-specific PMCA2 (_2 line) or PMCA3 (_3 line), which have been validated in our several other studies [36, 37]. The most critical finding was permanently increased resting cytosolic Ca^2+^ concentration in PMCA-reduced lines due to compromised Ca^2+^ extrusion ability observed even despite compensatory stimulation of PMCA1 expression detected in both lines and of PMCA4 in _3 line [36]. We have also provided the evidence that altered PMCA composition may play a role in regulation of bioenergetic function of mitochondria [37, 38]. Moreover, PMCA altered expression of genes encoding a number of elements responsible for regulation of calcium homeostasis [39, 40]. Taking into consideration that decreased amount and activity of PMCA may underlie many neurodegenerative diseases, here we analyzed whether the modified profile of plasma membrane calcium pumps can influence cell response to CCL5-induced signaling.

## 2. Materials and Methods

### 2.1. Cell Culture

Cell culture and differentiation PC12 rat pheochromocytoma cells (ATCC, USA) were routinely maintained in RPMI-1640 medium (Biowest, USA) containing 15% fetal bovine serum (Biowest, USA), 25 mM HEPES, pH 7.4 (21°C), 2 mM L-glutamine, 25 U/ml penicillin, and 25 *μ*g/ml streptomycin in a humidified incubator at 37°C with 5% CO_2_. Stable transfected cell lines with reduced PMCA2 (_2) or PMCA3 (_3) protein level were achieved using an antisense RNA cloned into pcDNA3.1(+) vector transfected to naive PC12 cells, as described previously [36]. Following selection with increasing G418 concentration (up to 1 mg/ml), the stable transfectants with nearly 50% reduction in PMCA2 or PMCA3 protein level were obtained. PC12 cells carrying an empty vector were used as a control (C). PC12 cells derived from a transplantable rat pheochromocytoma can differentiate into sympathetic-like neuronal cells upon exposure to neurotrophins, but similar effects can be triggered by db-cAMP. In our model differentiation process was induced with 1 mM dibutyryl-cAMP (Santa Cruz Biotechnology) for 48 h. Because PC12 cells exhibit some level of variability, routinely no more than 12 passages were used.

### 2.2. Ca^*2+*^ Measurement

Cells grown to 80% confluence in 25 cm^2^ flasks were detached from the surface and centrifuged at low speed (250 × g) for 5 min. 100 *μ*l of cell suspension was immediately transferred to 96-well plates. An equal volume of 2× concentrated Fluo-4 Direct calcium reagent loading solution (Life Technologies, USA) was added to each well and plates were incubated at 37°C for 1 h. Fluorescence was measured by using a Victor X3 plate reader (Perkin-Elmer). The measurements were performed for 250 s at one second intervals at 37°C and the fluorescence signal was recorded using 488 nm excitation filter and 535 nm emission filter. The signal was calibrated by addition of 0.1% Triton X-100 to obtain R_max_ and 10 mM EGTA to chelate extracellular Ca^2+^ to obtain R_min_. Appropriate controls for estimation of background fluorescence including phenol red free RPMI medium, Fluo-4 solution alone, and cell-free recording solution were included. Changes in fluorescence of Fluo-4 dye were converted to free cytosolic Ca^2+^ concentration according to the equation [Ca^2+^]_free_= K_d_([F-F_min_]/[F_max_-F]), where K_d_= 345 nM. All measurements were performed in a calcium-free buffer containing 125 mM NaCl, 5 mM KCl, 1.2 mM KH_2_PO_4_, 1.2 mM MgSO_4_, 6 mM glucose, and 25 mM HEPES. In all experiments, after measurement of basal Ca^2+^ for 150 s, 50 ng/ml of CCL5 (PeproTech) was added. For analysis of CCR/PLC/IP_3_R pathway, specific inhibitors were added right before measurements—cocktail of CCRs inhibitors: 1 nM BX513 (Abcam) for CCR1, 1 *μ*M SB328437 (Abcam) for CCR3 and 1 nM DAPTA (Abcam) for CCR5 or 4 *μ*M U73122 (Abcam) for PLC, or 100 *μ*M 2-APB (Sigma) for IP_3_Rs. To determine the role of ER calcium stores in CCL5 response, 1 *μ*M thapsigargin (Santa Cruz Biotechnology) was added 50 s after basal calcium measurement, and next 50 ng/ml of CCL5 at 150 s was applied. All measurements were done in duplicate and the data presented in the figures are the average of at least 5 independent cell cultures.

### 2.3. RNA Isolation and Real-Time PCR

Total cellular RNA was isolated using Tri Reagent (MRC) according to the manufacturer's protocol. 1 *μ*g of purified RNA and oligo(dT) primers were then used for cDNA synthesis using High-Capacity cDNA Reverse Transcription Kit (Applied Biosystems). The IP_3_R genes expression level was quantified in a real-time PCR reaction using 5x HOT FIRE Pol Eva Green qPCR Mix Plus (Solis Biodyne) in the following conditions: 15 min at 95°C followed by 40 cycles at 95°C for 15 s, 60°C for 30 s, and 72°C for 30 s using ABI Prism 7000 sequence detection system (Applied Biosciences). Following normalization to the expression of endogenous* Gapdh*, the fold change of each target gene was calculated using comparative 2^−ΔΔCt^ method as described previously [36]. Primers were synthesized in the Institute of Biochemistry and Biophysics (Poland) and their specificity was confirmed by running a melting curve after each reaction. Primers were designed using GenScript Primer Design Tool (USA) and their sequences were as follows:

Ip3r-1 forward: 5′-GTGGAGGTTTCATCTGCAAGC-3′,

  reverse: 5′-GCTTTCGTGGAATACTCGGTC-3′,

Ip3r-2 forward: 5′-GCTCTTGTCCCTGACATTG-3′,

  reverse: 5′-CCCATGTCTCCATTCTCATAGC-3′,

Ip3r-3 forward: 5′-AGCGAGAAGCAGAAGAAAG-3′,

  reverse: 5′-CATCCGTGGGAACCAGTC-3′,

Gapdh forward: 5′-GCTTACCAG-GGCTGCCTTCT-3′,

  reverse: 5′-CTTCCCATTCTCAGCCTTGAC-3′.

### 2.4. Western Blotting

Approximately 40-80 *μ*g of cell lysate proteins were separated using either 8% or 10% SDS-PAGE and electroblotted onto nitrocellulose membranes using semidry method. The membranes were blocked with 3% BSA in TBS buffer (10 mM Tris–HCl, pH=7.4, 150 mM NaCl) for 30 min at room temperature prior to exposure to primary antibodies. Membranes were incubated overnight at 4°C with the primary antibodies and for approximately 4 h with the secondary antibodies at room temperature followed by 3 × 15 min wash with TBS-T between each antibody application. The following primary antibodies were used: anti-CCR1 (rabbit, polyclonal, 1:1000, Santa Cruz Biotechnology; rabbit, polyclonal, 1:1000, BosterBio), anti-CCR3 (rabbit, polyclonal, 1:1000, Santa Cruz Biotechnology; rabbit, monoclonal, 1:1000, BosterBio), anti-CCR5 (mouse, monoclonal, 1:1000, Santa Cruz Biotechnology), anti-IP_3_R-1 (rabbit, polyclonal, 1:2000, Alomone Labs), anti-IP_3_R-2 (rabbit, polyclonal, 1:200, Alomone Labs), monoclonal anti-IP_3_R-3 (mouse, monoclonal, 1:2000, BD Biosciences), and anti-IP_3_R-1/2/3 (mouse, monoclonal, 1:500, Santa Cruz Biotechnology). Monoclonal anti-*β*-actin antibody (Santa Cruz Biotechnology) was used at 1:1000 dilution as a protein loading control. Secondary antibodies coupled with alkaline phosphatase (Sigma) at 1:5000 dilution were used for staining. Sigma Fast BCIP/NBT was used for visualization according to the manufacturer's instruction. Blots were scanned and band intensities were measured quantitatively using GelDoc™ EQ system with Quantity One 1-D Analysis Software version 4.4.1 (Bio-Rad).

### 2.5. Confocal Imaging

Cells were seeded at a density of 2 × 10^4^ cells/well on thin-glass bottomed 96-well plate (SensoPlate™, Greiner Bio-One) and differentiated for 48 h. Next, cell cultures were washed once with PBS and fixed for 10 min in room temperature with 2% formaldehyde freshly prepared from paraformaldehyde and buffered to pH = 7.4 in PBS. Subsequently, cells were washed once and further incubated for 1 h with the blocking buffer (10% normal goat serum, 0.1% Triton X-100, PBS pH = 7.4), following 1 h incubation with primary antibody against CCR5 diluted in the blocking buffer (1:100, Santa Cruz Biotechnology). Next, cells were washed 3 × 5 min with the blocking buffer and incubated for 1 h with secondary goat anti-mouse IgG1 antibody conjugated with Alexa Fluor 488 (1:1000, Thermo Fisher Scientific) to fluorescently label immunodetected CCR5. Subsequently, after 3 × 5 min wash with the blocking buffer, plasma membrane marker protein Na^+^/K^+^ ATPase was immunostained for 1 h with primary antibody (Abcam) fluorescently labeled with Zenon™ Alexa Fluor 568 Mouse IgG1 Labeling Kit (Thermo Fisher Scientific) and diluted in the blocking buffer (1:50). Finally, cells were washed 3 × 5 min with the blocking buffer, once with PBS, and were fixed again for 10 min in room temperature with 2% formaldehyde in PBS (pH = 7.4). Eventually, cell nuclei were counterstained with 5 *μ*M Hoechst 33342 in PBS, and triple-stained cells were visualized by confocal microscopy. Images were obtained using 780 LSM confocal microscope (Zeiss) with Plan-Apochromat 63x/1.4 Oil DIC M27 objective. ZEISS Microscope Software ZEN2012 was used to calculate mean fluorescence intensities of respective CCR5-positive pixels colocalizing with plasma membrane marker (Na^+^/K^+^ ATPase-positive) pixels. Plasma membrane marker-positive pixels were selected based on their fluorescence intensity that ought to be between arbitrary upper (500 units) and lower (40 units) thresholds, set either to eliminate nonspecific background staining or to negate the influence of high-intensity artificial staining (antibody precipitates and others). Analysis of fluorescence intensity of CCR5 staining colocalizing with plasma membrane staining was performed for each cell line for five different fields of view and results were averaged.

### 2.6. Data Analysis

The data are shown as means ± SD of n separate experiments (n ≥ 3) with the exact n value given under each figure. The comparisons between cell lines were done using Student's* t*-test or ANOVA followed by Tukey or Bonferroni's multiple comparisons tests, wherever applicable. P values were calculated using STATISTICA 12.0 (StatSoft) or GraphPad Prism 7 (GraphPad, La Jolla, CA). P < 0.05 was considered as statistically significant.

## 3. Results

### 3.1. CCRs Expression in PC12 Cell Lines

**Figure 1:**
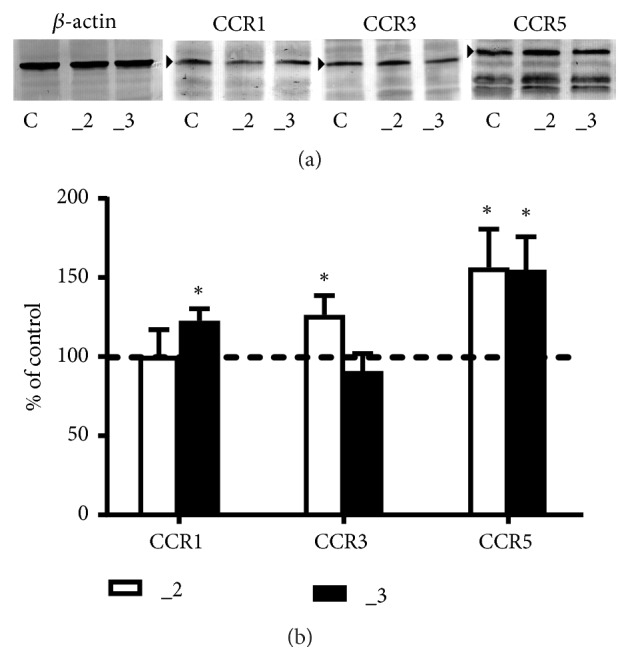
Western blot analysis of CCRs protein in PC12 cell lines. (a) Approximately 40–60 *μ*g of total protein was resolved on a 10% SDS-PAGE gel and electroblotted onto nitrocellulose membranes. Membranes were probed with polyclonal anti-CCR1, polyclonal anti-CCR3, or monoclonal anti-CCR5. Representative blots are shown and arrows indicate the main band of the receptors with apparent MW 41 kDa for CCR1, 40 kDa for CCR3, and 46 kDa for CCR5. (b) The bands intensity was densitometrically analyzed and the results are expressed as % of the control PC12 cells obtained after normalization to endogenous *β*-actin level (± SD). *∗* P < 0.05 (n=7), C: control line, _2: PMCA2-reduced line, _3: PMCA3-reduced line.

First, we evaluated the presence of CCR1, CCR3, and CCR5 in PC12 lines using two approaches. The immunoreactivity of examined receptors was initially assayed in a cell lysate by Western blot (Figure 1). In comparison to the control cells, CCR1 was present at higher level in _3 cells, CCR3 increased in _2 cells, but in both PMCA downregulated PC12 lines CCR5 amount increased by nearly 50%.

**Figure 2:**
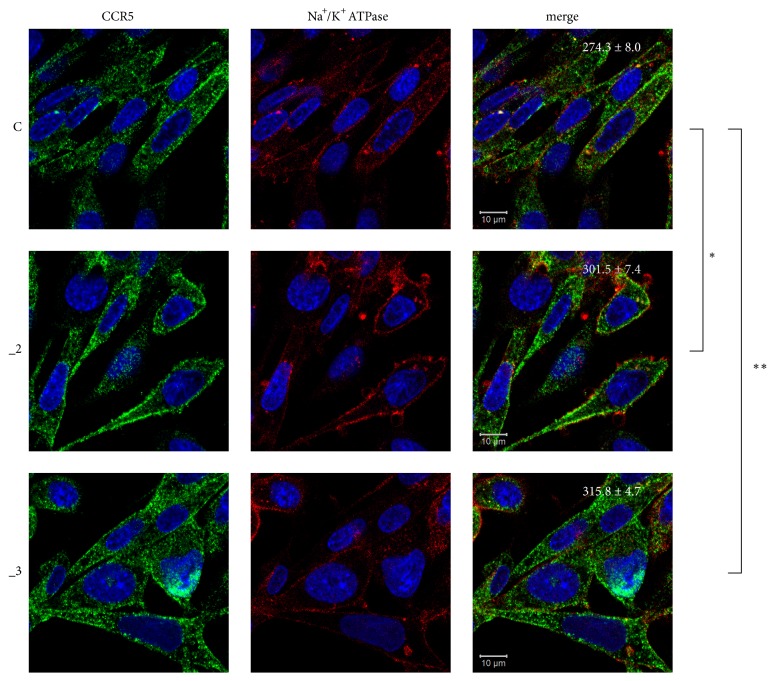
Localization of CCR5 in PC12 cell lines. PC12 cells differentiated for 48 h with db-cAMP were fixed and immunostained with antibodies against CCR5 receptor (green) and Na^+^/K^+^ ATPase (plasma membrane marker, red). Nuclei were stained with Hoechst 33342 (blue). Representative confocal images are presented. Values shown in merged images represent average fluorescence intensity ± SEM (n = 5) of pixels positive in green channel (CCR5) that colocalize with red channel positive pixels (Na^+^/K^+^ ATPase). *∗* P< 0.05, *∗∗* P < 0.01. Scale bars: 10 *μ*m. C: control line, _2: PMCA2-reduced line, _3: PMCA3-reduced line.

Since functionally active chemokine receptors are located at the plasma membranes, we next explored cellular distribution of CCRs by confocal microscopy. Staining of endogenous CCR1 and CCR3 showed low immunosignal intensity, which was concentrated predominantly in the cytosolic area (data not shown). To validate these results, we repeated the assay using another set of antibodies against CCR1 and CCR3; however, we obtained similar results. These data indicated that although CCR1 and CCR3 were present in PC12 cell lines, their participation in CCL5 signaling in our model seems to be negligible. CCR5 was expressed much more abundantly and was mostly distributed in the plasma membranes (Figure 2). Quantification of CCR5 immunofluorescence revealed that cells with reduced PMCA2 or PMCA3 contained more plasma membrane-located receptor than that of control line.

### 3.2. CCL5 Effect on Calcium Transients

**Figure 3:**
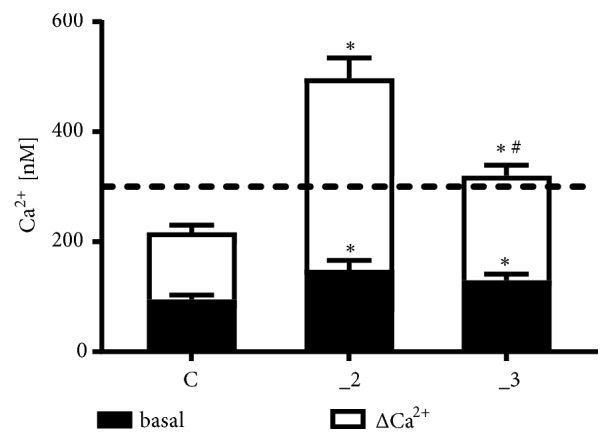
CCL5 effect on Ca^2+^ release in PC12 cell lines. The maximal level of [Ca^2+^]c liberated by 50 ng/ml CCL5 was reached approximately during first seconds after addition of chemokine and was determined as an average of peak values from 5 separate experiments (± SD). Δ[Ca^2+^]c was calculated by subtracting the resting level (black box) from maximal Ca^2+^ concentration. *∗* P < 0.05 vs. control line, ^#^ P < 0.05 _3 line vs. _2 line (n=5). _2: PMCA2-reduced line, _3: PMCA3-reduced line.

To analyze CCL5-induced cytosolic Ca^2+^ increase in examined PC12 cell lines, chemokine action was assayed in a calcium-free medium to avoid the secondary effect triggered by “calcium-induced calcium release” process, as well as the concomitant participation of membrane calcium channels. Changes in fluorescence were monitored for 250 s at one-second intervals, and CCL5 was always applied after 150 s of measurement. The CCL5 concentration was chosen because it was shown to be effective under inflammatory state, which is supported by literature data. It is also frequently used for* in vitro* assays including PC12 cells [41]. At resting state higher Ca^2+^ level in PMCA-reduced PC12 cells was observed (Figure 3), confirming our previous results [36].

**Figure 4:**
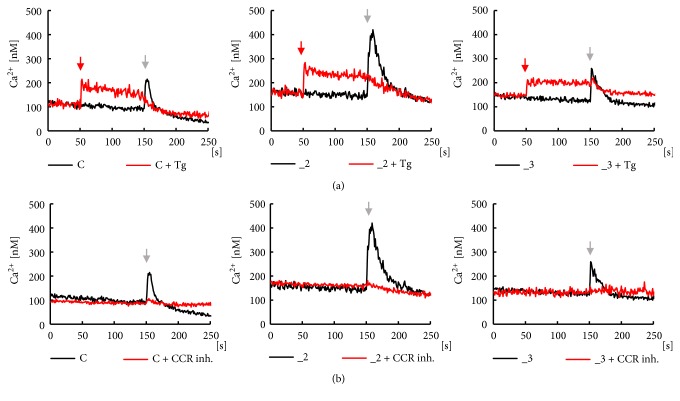
Analysis of CCL5 effect on calcium transients in PC12 cell lines. Ca^2+^ transients were measured in parallel wells with (red lines) or without (black lines) presence of specific inhibitors. (a) SERCA inhibitor, 1*μ*M thapsigargin, was added after 50 s (red arrow), and next 50 ng/ml CCL5 was applied after 150 s (black arrow). (b) CCRs inhibitors, 1 nM BX513 for CCR1, 1 *μ*M SB328437 for CCR3, and 1 nM DAPTA for CCR5, were included just before measurements. 50 ng/ml CCL5 was applied after 150 s (black arrow). All measurements were done in duplicate and the presented traces are average from 5 independent cell cultures (n=10). C: control line, _2: PMCA2-reduced line, _3: PMCA3-reduced line.

Treatment with CCL5 at 50 ng/ml increased cytosolic Ca^2+^ in all examined lines, more intensively in PMCA-modified cells, but the highest peak value was observed in _2 line. This was also confirmed by analysis of area under the curves. Time course of chemokine effect presented in Figure 4(a) (black lines) showed a fast transient increase in [Ca^2+^]_c_ during first seconds after CCL5 application, followed by a slow, progressive decrease in [Ca^2+^]_c_. The estimated time required for recovery of Ca^2+^ to the basal level was 14 ± 1 s for control PC12 cells, 41 ± 8^*∗*^ s for _2 line, and 23 ± 7^*∗*#^ s for _3 line (n=5; *∗*P < 0.05 vs. control; ^#^P < 0.05 _3 vs. _2). Slower recovery in _2 and _3 lines could result from lowered Ca^2+^-clearing potency in PMCA-reduced lines, since SERCA affinity for calcium is at low micromolar range [42]. In Ca^2+^-free conditions, the differences in cell response to CCL5 might reflect a balance between intensity of Ca^2+^ release from ER and Ca^2+^ extrusion, which varied between cell lines.

To verify that in our experimental conditions the endoplasmic reticulum was a main source of calcium, the assay was performed in the presence of 1*μ*M thapsigargin added after 50 s, and next CCL5 was applied at 150 s. As shown in Figure 4(a) (red lines), Tg caused a successive calcium depletion from ER due to inhibition of Ca^2+^ reuptake by SERCA, and subsequent addition of chemokine had no effect on calcium transient. To confirm the participation of CCRs in generation of Ca^2+^ transient, the assay was also performed in the presence of specific CCRs inhibitors. Although CCR5 appeared to be the main functionally active receptor in our cell lines, chemokine action was assessed in the presence of cocktail of CCR1, CCR3, and CCR5 inhibitors added just before measurement (Figure 4(b), red lines). CCL5 applied after 150 s did not affect Ca^2+^ level indicating that examined CCRs were indeed the crucial targets for the chemokine.

### 3.3. Analysis of PLC/IP_3_R Pathway

**Figure 5:**
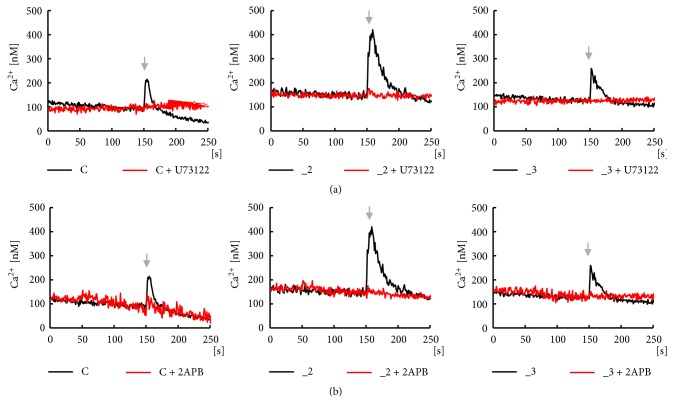
Analysis of PLC/IP_3_R signaling pathway in PC12 cell lines. Analysis of PLC/IP_3_R pathway was performed in parallel wells with (red lines) or without (black lines) presence of specific inhibitors included just before measurements. 50 ng/ml CCL5 was always applied after 150 s (black arrow). The calcium transients were assayed in the presence of (a) PLC inhibitor, 4*μ*M U73122, and (b) IP_3_Rs inhibitor, 100 *μ*M 2-APB. All measurements were done in duplicate and the presented traces are average from 5 independent cell cultures (n=10). C: control line, _2: PMCA2-reduced line, _3: PMCA3-reduced line.

In the next step we analyzed the downstream signaling triggered by CCL5/CCR complex by inhibiting the crucial elements of this pathway, PLC and IP_3_ receptors. As shown in Figure 5(a) (red lines), in the presence of selective PLC inhibitor, U73122, there was no Ca^2+^ response to CCL5 in examined cell lines, which confirmed that PLC-induced signaling must be the chemokine effector. Subsequent generation of a second messenger, IP_3_, leads to the opening of IP_3_-dependent calcium channels. Thus, we treated cells with 2-APB, an inhibitor of IP_3_ receptors (Figure 5(b), red lines). Baseline Ca^2+^ level was not affected by the chemokine in these conditions, showing that IP_3_Rs were indeed responsible for observed calcium transients.

### 3.4. Characteristics of IP_*3*_ Receptors in PC12 Cell Lines

**Figure 6:**
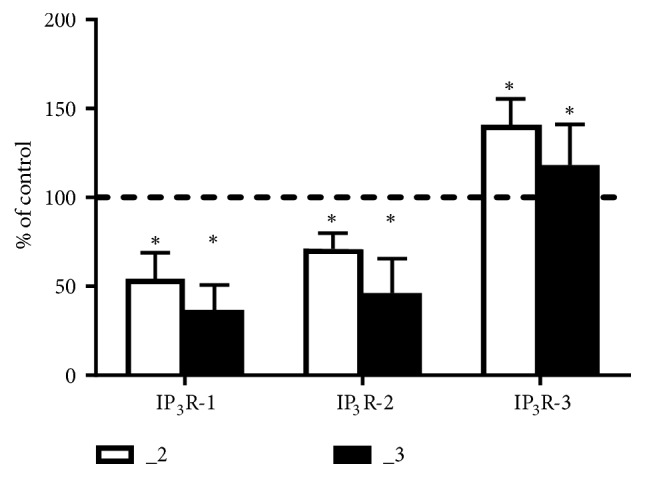
Real-time PCR analysis of IP_3_ receptors in PC12 cell lines. A relative fold change (± SD) obtained following normalization to* Gapdh* expression and calculated using comparative 2^−ΔΔCt^ method. The expression of a target gene in control PC12 cells was taken as 100% and is presented as a dotted line. Statistical differences from values in control are indicated by *∗*P < 0.05 (n = 6). C: control line, _2: PMCA2-reduced line, _3: PMCA3-reduced line.

Since IP_3_ receptors appeared to be the final CCL5 effectors in analyzed pathway, we next characterized their expression in our PC12 cell lines. Real-time PCR analysis showed significant IP_3_R-1 and IP_3_R-2 downregulation in both _2 and _3 lines, whereas the expression of IP_3_R-3 increased in comparison to the control cells (Figure 6).

**Figure 7:**
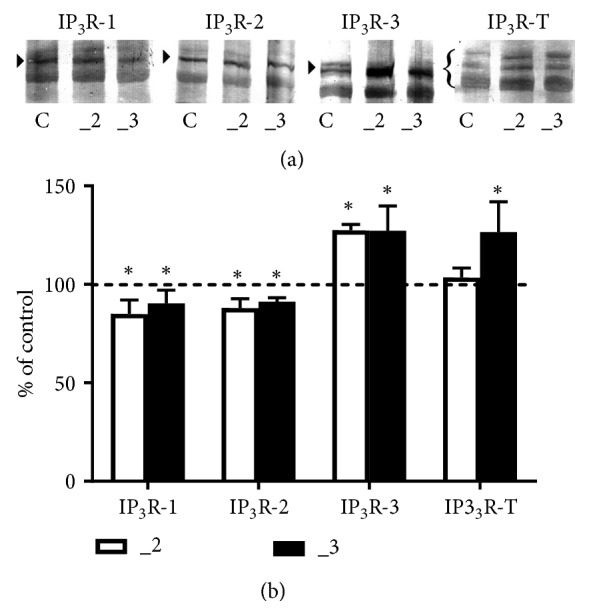
Western blot analysis of IP_3_ receptors in PC12 cell lines. (a) Approximately 60–80 *μ*g of total protein was resolved on an 8% SDS-PAGE gel and electroblotted onto nitrocellulose membranes. Membranes were probed with anti-IP_3_R1, anti-IP_3_R2, anti-IP_3_R3, and recognizing all isoforms anti-IP_3_R-T antibodies. Representative blots are shown and arrows indicate the main band of the receptors. (b) Band intensity was densitometrically analyzed and the results are expressed as % (± SD) of control PC12 cells obtained after normalization to endogenous *β*-actin level. *∗*P< 0.05 (n=7). C: control line, _2: PMCA2-reduced line, _3: PMCA3-reduced line.

To validate whether the mRNA changes correlated with corresponding receptor protein, Western blot analysis was performed using isoform-specific antibodies and the antibody that recognized all three IP_3_ receptors (Figure 7(a)). Single bands of the predicted sizes for IP_3_-1, 2, and 3 receptors (~313 kDa, 260 kDa, and 250 kDa, respectively) were detected in all lines, but some lower molecular weight bands were also present. They could represent proteolytic fragments of the receptors, as similar observations were also shown in another study [43].

Quantification of individual IP_3_R isoforms confirmed that in _2 and _3 lines the protein level of IP_3_R-1 and 2 was lower than that of control cells, but IP_3_R-3 increased by ~40% (Figure 7(b)). However, analysis of blots probed with antibody recognizing all IP_3_ receptors revealed a total increase only in _3 line. This apparently indicates the differences in a ratio between IP_3_R isoforms and finally could result in diversified affinity for IP_3_ in both PMCA-reduced cell lines.

## 4. Discussion

Our previous studies revealed that, in differentiated PC12 cells, downregulation of neurospecific PMCA2 or PMCA3 increased cytosolic Ca^2+^ and subsequently affected the expression level of several Ca^2+^-associated proteins, i.e., SERCA, calmodulin, calcineurin, neuromodulin (GAP43), and certain types of voltage-gated calcium channels [36, 39, 40]. We have also revealed a compensatory increase of PMCA1 isoform in both PMCA-reduced lines coexisting with higher expression of SERCA2 and SERCA3, which correlated with higher Ca^2+^ accumulation in the endoplasmic reticulum [36]. On the one hand it could effectively decrease cytosolic Ca^2+^ concentration to its safe level, but on the other hand, it may potentially increase Ca^2+^ release after activation of calcium channels in the ER, including activation of IP_3_ receptors and ryanodine receptors [42]. Since IP_3_R-mediated Ca^2+^ release from the ER and mitochondrial Ca^2+^ homeostasis are physiologically coupled, their improper cooperation may significantly affect cell viability [44].

Based on current study, we can add to the list of common features detected after reduction of PMCA2 or PMCA3 increased level of CCR5 and IP_3_R-3 proteins, but lowered IP_3_R-1 and IP_3_R-2. All these changes are primarily related to elevated cytosolic Ca^2+^ as a consequence of changed composition of PMCAs. Interestingly, some of them could occur as adaptive processes protecting cells against calcium overload. Since age-related PMCA decrease has been documented [17, 45–47], our modified PC12 cells may be a useful model to clarify the biological changes in neurons associated with aging and, potentially, to study the vulnerability of cells to neurodegenerative insults.

The presence of CCR5 in PC12 cells was shown in several studies [25, 41], and it was also confirmed by our data. Moreover, we detected CCR1 and CCR3 proteins, but their cytosolic localization suggested that CCL5 signal was mainly transmitted by CCR5, because the activity of receptor requires its presence in the plasma membrane. Upregulation of CCR5 has been revealed in a number of neurological disorders and models of CNS injury, where it is often localized in astrocytes and microglial cells [48–51]. Overactivation of CCR5 with subsequent raise of cytosolic Ca^2+^ affected chemotaxis, secretion, and gene expression and could lead to inflammatory and degenerative processes in the CNS [27, 29]. Chemokine receptors could bind several chemokines and could act as multimeric forms, homo- or heterodimers [52–55]. The additional mechanism that may modify CCL5 signaling depends on the function of another type of calcium channels existing in ER, ryanodine receptors. RyRs are activated by a mechanism known as Ca^2+^-induced Ca^2+^ release (CICR) and involve cooperation with plasma membrane calcium channels [56]. Crucial for RyRs function is a high, micromolar Ca^2+^ concentration necessary to open these channels [57]. In our study, to avoid the potential influence of RyR-mediated secondary effects, we assayed CCL5 action without external calcium.

Using the selected inhibitors we confirmed that in our cell models CCL5 downstream effects involved CCR5-PLC-IP_3_R pathway. The crucial step appeared to be associated with activation of IP_3_ receptors. Although IP_3_R-1 represents a predominant isoform in the central nervous system, other isoforms also exist in some brain areas and may differ in neuronal compartmentalization [58–60]. A particular role of IP_3_Rs in hippocampus is related to learning and memory abilities, and changes in IP_3_R isoforms composition during aging may have an impact on increased deficits in these processes. In most cultured cell types IP_3_R-3 is the principal form, but IP_3_R-1 and IP_3_R-2 have also been detected [61]. All isoforms exhibit specific characteristics: IP_3_R-1 possesses low Ca^2+^ affinity and medium affinity for IP_3_, IP_3_R-2 represents the isoform with the highest affinity for both Ca^2+^ and IP_3_, and IP_3_R-3 is the most sensitive for modulation by Ca^2+^, but displays the lowest IP_3_ affinity [60]. Interestingly, IP_3_Rs are regulated in a biphasic way by cytosolic Ca^2+^ and are stimulated at low Ca^2+^ level, but inhibited by higher than 300 nM Ca^2+^ [33]. Up to now, over 100 proteins have been identified to interact with and regulate the IP_3_Rs [62]. Thus, multiple regulatory processes for each IP_3_R isoform may produce diversified cell signaling paths that can initiate the adaptive response or can lead to neurodegeneration [60, 61].

**Figure 8:**
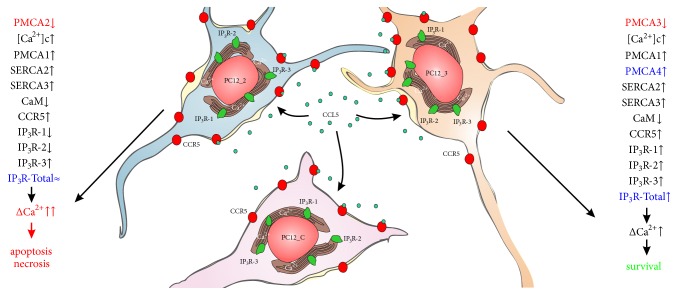
Schematic presentation of CCL5 effect on PC12 cells. Downregulation of neurospecific PMCA2 or PMCA3 isoforms in differentiated PC12 cells generated two types of cell response: similar for both lines or characteristic for only one line. The common changes were increased cytosolic Ca^2+^ and, as a compensatory mechanism, upregulation of PMCA1 isoform, enlarged expression of SERCA2 and SERCA3, and diminished calmodulin amount [36, 40]. The levels of CCR5 and IP_3_R-3 proteins also increased, but the expression of IP_3_R-1 and IP_3_R-2 was lowered (present study). Interestingly, altered IP_3_R isoform composition did not change total IP_3_R protein in the _2 line, while it increased in the _3 line. Also in _3 cells, the amount of PMCA4 increased [36]. These subtle differences could have profound consequences after CCL5/CCR5 activation, since potency to restore the basal Ca^2+^ level in the _3 line appears to be higher than in the _2 line, which may be essential for the survival of the cell. Under prolonged Ca^2+^ signal in the _2 line due to reduction of the fastest isoform - PMCA2, the subsequent Ca^2+^-mediated processes could increase vulnerability to cell death. Abbreviations used: CaM, calmodulin; CCL5, chemokine C-C motif ligand 5; CCR5, receptor for CCL5; inositol 1,4,5-triphosphate (IP_3_); IP_3_R, IP_3_ receptor; PMCA, plasma membrane Ca^2+^-ATPase; SERCA, sarco/endoplasmic Ca^2+^-ATPase.

Our analysis showed that in PC12 cells, all three IP_3_R isoforms were present, with IP_3_R-3 being the most prominent subtype. An interesting observation made here was the altered composition of IP_3_Rs in PMCA-reduced lines at both mRNA and protein level. IP_3_R-1 and IP_3_R-2 decreased, whereas IP_3_R-3 amount was higher than that of control cells. Changes in relative mRNA level indicate that the regulation occurs at the level of transcription, suggesting Ca^2+^-dependent negative feedback loop for IP_3_R-1 and IP_3_R-2. Analysis of IP_3_R protein levels also confirmed the differences in a ratio of IP_3_R isoforms between control _2 and _3 lines. One can assume that in both PMCA-reduced cells the decreased expression of receptors with higher affinity for IP_3_ could lower the total apparent sensitivity to IP_3_ and thereby may provide some protection against calcium overload. The graphical summary of these findings is presented in Figure 8.

In _2 line, we observed higher Ca^2+^ accumulation in the ER; thus potentially more calcium could be released after activation of IP_3_Rs. Moreover, less efficient PMCA-dependent extrusion system, due to PMCA2 suppression, may prolong calcium signaling, modifying a number of existing pathways. It can also lead to further Ca^2+^ overload. In fact, in this line we reported decreased cell survival and increased percentage of apoptotic cells [36]; thus the cells may exhibit higher vulnerability to calcium-induced cytotoxicity. Correlation between PMCA2 and disturbances in cell function resulting in augmented cell death has been reported in neurons, indicating a protective PMCA2 role [13, 63]. Taking into account the fact that PMCA2 represents nearly 40% of the total pump in the brain [64], reduction of PMCA2 appears to be more harmful for the cells than deficiency of PMCA3, and compensatory mechanisms may not be sufficient for full protection of cells.

The presence of an additional protective mechanism may be suggested in the _3 line, where we previously observed increased expression of PMCA4 isoform. One of the well-established mechanisms of PMCA regulation is stimulatory action of PIP_2_ [9, 65, 66]. Recently, a new look on PMCA/PIP_2_ interaction has been proposed [67]. Binding of PIP_2_ by PMCA4 has been demonstrated to protect plasma membrane PIP_2_ from hydrolysis by PLC. It could potentially limit IP_3_ production and, subsequently, restrict Ca^2+^ release from ER. Since for full activity, IP_3_Rs must bind four IP_3_ molecules [68], a sufficient available PIP_2_ concentration as a substrate for PLC is a necessary requirement. Accordingly, in the _3 line greater protection could result not only from the altered profile of IP_3_Rs and the presence of highly active PMCA2, but also from higher PMCA4 amount. All these adaptive changes may limit the amount of Ca^2+^ released from the ER and shorten the time necessary to restore basal Ca^2+^ level.

## 5. Conclusions

Downregulation of neuron-specific PMCA2 or PMCA3 initiated a set of responses that significantly altered Ca^2+^-induced signaling that may allow cells to survive but also may promote cell death. The unique role played by “fast” isoforms of plasma membrane calcium pump in neuronal cells suggests that long-lasting calcium dyshomeostasis could markedly increase cell vulnerability to pathological events, including neurodegenerative disorders [13, 19, 63]. Aberration in the activity of PMCA2 has been implicated in some diseases, i.e., Huntington's disease and multiple sclerosis, but altered expression of PMCA3 has been linked with cerebellar ataxia [69]. Here we demonstrated that CCL5/CCR5 signaling could be one of the critical mediators of the cell fate. Although we analyzed only the initial step in CCL5-induced pathway, this key phase could play a decisive role for the cell. In the aging brain increased BBB permeability, augmented leukocyte infiltration, and more severe CCL5 action under less efficient neuronal Ca^2+^ extrusion mechanism could accumulate potentially harmful changes in neurons, which increase the risk of developing neurodegenerative diseases.

## Data Availability

All of data used to support the findings of this study are included within the article.
